# Retention in a NGO Supported Antiretroviral Program in the Democratic Republic of Congo

**DOI:** 10.1371/journal.pone.0040971

**Published:** 2012-07-17

**Authors:** Olivier Koole, Lucien Kalenga, Modeste Kiumbu, Joris Menten, Robert W. Ryder, Henri Mukumbi, Robert Colebunders

**Affiliations:** 1 Institute of Tropical Medicine, Antwerp, Belgium; 2 AmoCongo, Lubumbashi, Democratic Republic of Congo; 3 School of Public Health, Kinshasa, Democratic Republic of Congo; 4 Division of Hospital Medicine, University of California San Diego, San Diego, California, United States of America; 5 University of Antwerp, Antwerp, Belgium; Tulane University, United States of America

## Abstract

**Background:**

Retention of patients in ART care is a major challenge in sub-Saharan programs. Retention is also one of the key indicators to evaluate the success of ART programs.

**Methods and Findings:**

A retrospective review of 1500 randomly selected medical charts of adult ART patients from a local non-governmental (NGO) supported ART program in the Democratic Republic of Congo (DRC). Retention was defined as any visit to the clinic in the 4 months prior to the abstraction date. Retention over time and across different sites was described. The relationship between patient characteristics and retention rates at 1 year was also examined. 1450 patients were included in the analysis. The overall retention rates were 81.4% (95% CI: 79.3–83.4), 75.2% (95% CI: 72.8–77.3), 65.0% (95% CI: 62.3–67.6) and 57.2% (95% CI: 54.0–60.3) at 6 months, 1 year, 2 years and 3 years respectively. The retention rates between sites varied between 62.1% and 90.6% at 6 months and between 55.5% and 86.2% at 1 year. During multivariable analysis weight below 50 kg (aHR: 1.33, 95%CI: 1.05–1.69), higher WHO stage at initiation (aHR: 1.22, 95%CI 0.85–1.76 for stage 3 and aHR: 2.98, 95%CI: 1.93–4.59 for stage 4), and male sex (aHR: 1.32, 95%CI: 1.05–1.65) remained as significant risk factors for attrition during the first year after ART initiation. Other independent risk factors were year of initiation (aHR: 1.73, 95%CI: 1.26–2.38 for the year 2007 and aHR: 3.06, 95%CI: 2.26–4.14 for the period 2008–2009), and site.

**Conclusions:**

Retention is a major problem in DRC, while coverage of patients on ART is still very low. With the flattening of funding for HIV care and treatment in sub-Saharan Africa, and with decreasing funding worldwide, maximizing retention during the much needed scaling-up will even be more important.

## Introduction

Access to antiretroviral therapy (ART) continues to expand rapidly, especially in Sub-Saharan Africa. At the end of 2009 about 5.25 million people were receiving ART worldwide, and ART coverage reached about 36%. [1] However long term retention of patients in ART care remains a major challenge in sub-Saharan programs. [2] Results from a meta-analysis showed that only 75% of patients started on ART were still in care after 1 year, and about 61% after 2 years. [2] The major causes of non-retention (or attrition) are death and loss to follow-up (LTFU). [2]–[4].

Retention is also one of the key indicators to evaluate the success of ART programs. [5].

Improved retention in HIV programs is also one of the goals of the Treatment 2.0 initiative. [6] The Treatment 2.0 initiative, aims at further scaling up treatment over the next decade while maximizing the efficiency and effectiveness of HIV treatment.

The reported HIV-prevalence in the Democratic Republic of Congo (DRC) was 1.3% in 2007. [7] Antiretroviral therapy was introduced in 2005, and in 2009 about 35,000 patients were receiving ART. [1].

There are currently no data available on retention in ART programs in the Democratic Republic of Congo (DRC). The objective of this study was to estimate the level of retention of patients in the AmoCongo ART program across different sites in the Democratic Republic of Congo (DRC). The study also examined the relationship between patient characteristics and retention rates.

## Methods

### Design and Settings

AmoCongo is a Congolese non-governmental organization (NGO), that began in 1993 with providing support for orphans and vulnerable children in Kinshasa. Over the years the NGO expanded its activities to several provinces and included voluntary counseling and testing, and provision of HIV care to adults.

In 2005 AmoCongo started with the provision of ART care in their Ambulatory Treatment Centers (ATCs), which are standalone clinics that offer counseling and testing, and HIV care. At the moment of the study AmoCongo received substantial funding from abroad (GFATM), which enabled them to use a doctor-based approach with assistance of registry clerks, counselors, and nurses, and with regular laboratory monitoring and free OI drugs and ART.

These centers have played a major role in the scaling up process of ART in the DRC. At the end of 2009, AmoCongo had ATCs in 27 health zones and had about 11,000 patients on ARVs, about one third of the patients on ART in the DRC at that moment.

**Figure 1:**
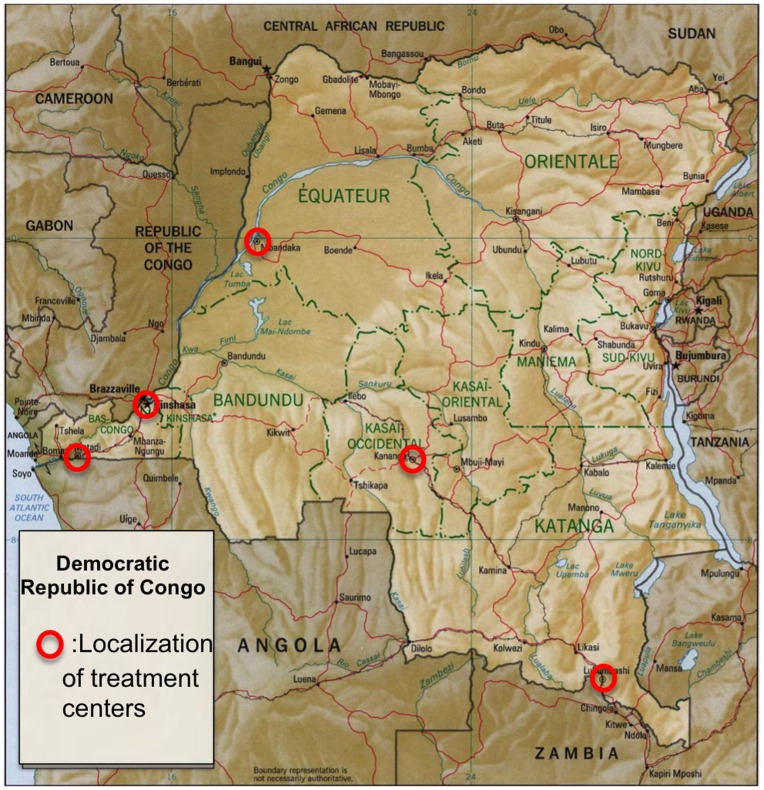
Map of Democratic Republic of Congo with localization of the study sites.

A retrospective cohort study of patients started on ART in the period 2005–2009 was conducted in the Democratic Republic of Congo (DRC) at six AmoCongo ART treatment centers. To ensure a balanced geographical spread study, sites were selected from different provinces: Kinshasa Province (Ndjili and Kasavubu), Katanga Province (Lubumbashi), Bas-Congo Province (Matadi), Kasaï-Occidental province (Kananga) and Equatorial Province (Mbandaka ) (**Figure 1**). Only sites with at least 300 patients on ART at the moment of data abstraction were considered for inclusion in the study.

### Inclusion Criteria

Patients who were at least 18 years of age at ART initiation and who initiated ART treatment (combination of 3 ARVs) at least six months prior to the data collection were included in the study. Patients who started ART before 1^st^ of January 2005 were excluded.

**Figure 2:**
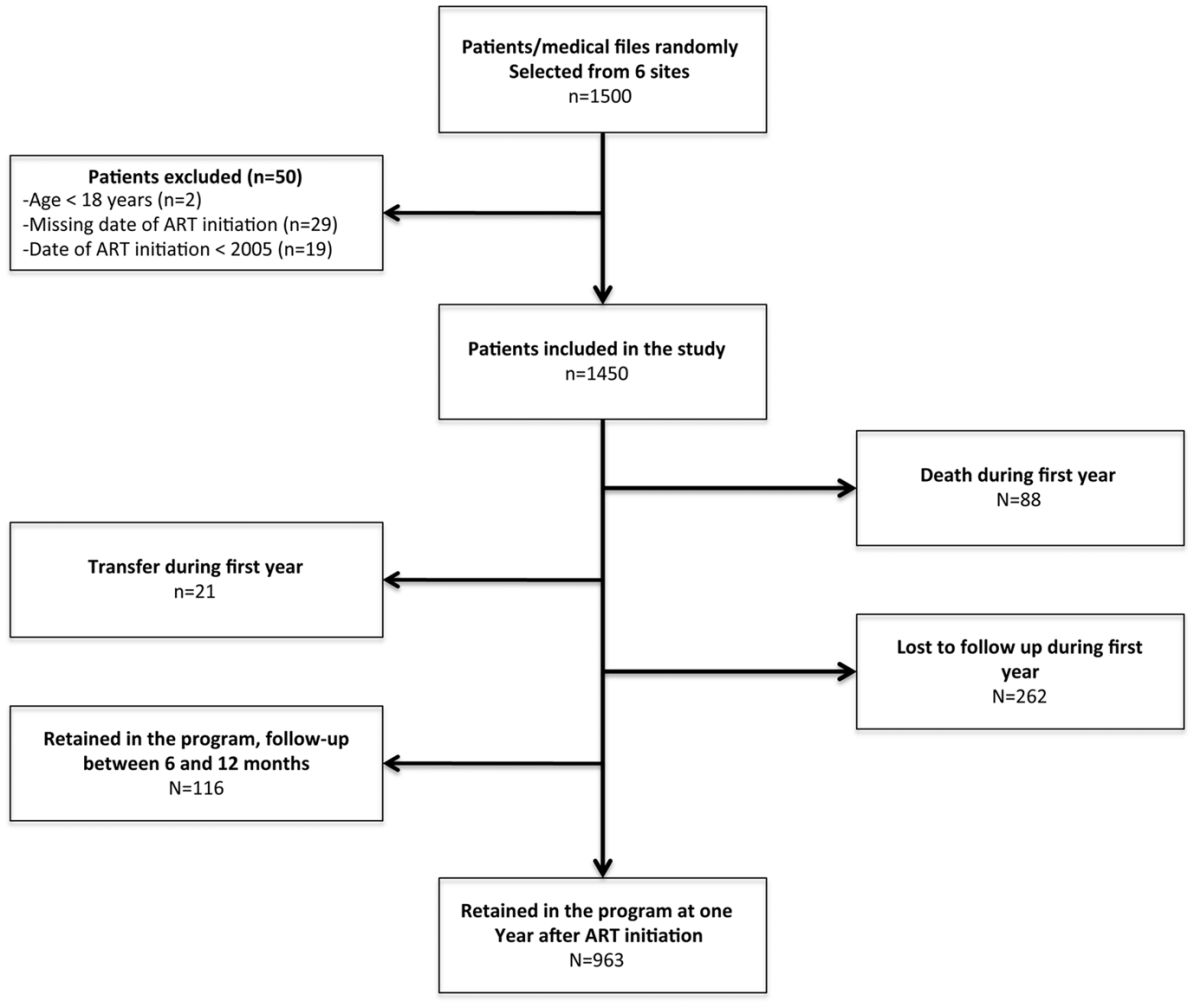
Flow chart of the study process.

### Data Collection

During the period September-December 2009 a retrospective chart review of 1500 medical charts was conducted. In each of the six research sites a random sample of 250 medical charts was selected from a sampling frame that consisted of all patients ever started on ART at that site. The sampling frame was prepared by the site supervisor; the list of the medical files to be selected was generated by the data analyst using a computerized random number generator (‘sample’ function in Stata). Randomly selected patients that were not eligible for the study were replaced by the next patient in the sampling frame.

Data abstraction was done by a team of 3 trained data abstractors per site and the site supervisor.

**Table 1:** Patient characteristics at ART initiation.

Characteristic	N = 1450
**Year of ART initiation:** n (%)	
2005–2006	458 (31.6)
2007	461 (31.8)
2008–2009	531 (36.6)
**Gender:** n (%)	
Male	491 (33.9)
Female	959 (66.1)
**Age (years):** median (range)	40 (18–74)
Age <40 years: n (%)	680 (46.9)
Age ≥40 years: n (%)	738 (50.9)
Missing: n (%)	32 (2.2)
**Civil status:** n (%)	
Single	194 (13.4)
Married	675 (46.6)
Widowed/divorced	560 (38.6)
Missing	21 (1.4)
**Weight (kg):** mean (SD)	56.9 (13.6)
Weight <50: n (%)	430 (29.7)
Weight ≥50: n (%)	858 (59.2)
Missing: n (%)	162 (11.2)
**CD4 (cells/µL):** median (IQR)	150 (71–238)
CD4<50: n (%)	85 (5.9)
CD4≥50<200: n (%)	258 (17.8)
CD4≥200: n (%)	182 (12.6)
Missing: n (%)	925 (63.8)
**TLC (cells/µl):** median (IQR)	1204 (840–1728)
TLC <1200: n (%)	372 (25.7)
TLC ≥1200: n (%)	74 (5.1)
Missing: n (%)	1,004 (69.2)
**Prior ART experience:** n (%)	
Yes	169 (11.7)
No	1,257 (86.7)
Missing	24 (1.6)
**WHO stage at start:** n (%)	
WHO stage 1–2	247 (17.0)
WHO stage 3	847 (58.4)
WHO stage 4	129 (8.9)
Missing	227 (15.7)
**Regimen at initiation:** n (%)	
D4T-3TC-NVP	1214 (83.7)
ZDV-3TC-NVP	104 (7.2)
D4T-3TC-EFV	76 (5.2)
ZDV-3TC-EFV	51 (3.5)
Other	5 (0.3)
**Distance from clinic:** n (%)	
<1 hour	644 (44.4)
≥1 hour <2 hours	186 (12.8)
≥2 hours	111 (7.7)
Missing	509 (35.1)

### Data Management and Analysis

A random sample of 250 ART-treated patients in each site was needed to allow the estimation of the retention rate to be measured with a precision of 5%, assuming the retention rate was at least 80%.

In the AmoCongo program, patients receive a supply of ART for 1 up to 3 months (depending on their clinical status, adherence and external factors as distance to the clinic). Retention in the ART program was defined as any visit to the clinic (for a clinical visit, or laboratory monitoring, or for drug refill at the pharmacy) in the 4 months prior to the abstraction date.

Retention rates overall and in each site were estimated using survival analysis methods: Kaplan-Meier survivor curves were constructed with 6 months, 1 year, 2 year and 3 year retention rates. The time origin was the date of ART initiation. Conform with the major causes of non-retention (or attrition) and published literature [2], [4], [8], the primary outcome was defined as death or loss to follow-up. As in the AmoCongo program some patients on ART received up to 3 months of supply of ART, the event of interest was defined as a reported death or no visit to the clinic in the 4 months prior to the abstraction date while not being transferred out. Patients retained in the program or patients who were transferred out were censored at their last visit. For patients who were reported dead (by the relatives or caregivers), the date of death was used to determine the time in the program, for patients who were considered not retained in the program (no visit in the 4 months prior to the abstraction date) the date of the last visit was used.

**Table 2:** Site characteristics at time of data abstraction.

Site	Kasavubu	Ndjili	Lubumbashi	Matadi	Kananga	Mbandaka
**Number of patients in** **the analysis** (N = 1450)	236	231	246	248	245	244
**Type of site**	urban	urban	urban	urban	semi-urban	semi-urban
**Number of patients on ART**	2713	1015	1495	1593	577	468
**Number of consultations** **per day**	80–100	30–40	40–50	30–40	20–30	20–30
**Number of providers**	25	10	15	14	8	8
**Patient medical record** **system**	electronic	electronic	electronic	electronic	manual	manual
**Number of supervisions** **during the last 6 months**	6	6	4	4	2	1
**CD4 availability at site**	yes	yes	yes	no*	no	no
**CD4 cell count**						
Median	148	126	141	189	NA	NA
IQR	68–230	62–234	64–204	100–299	NA	NA
Missing	127	78	97	151	NA	NA

* Done at a private clinic in Matadi.

Consistent with the majority of studies reporting on risk factors for mortality and attrition [9]–[12] multivariable Cox-regression models were used to describe the associations between attrition during the first 1 year after ART initiation and individual characteristics. The predictor analysis was limited to 1 year after ART initiation because attrition is highest during this period [13], and because the association between baseline values and the outcome wanes after this period. Predictor variables evaluated were age, gender, civil status, prior ART experience at another site, weight (instead of BMI because height was not available in the medical files), WHO clinical stage and CD4 count at start of ART, distance from the clinic, the ART regimen (NVP or EFV based) and the year ART began. Variables associated with retention with a p-value ≤0.10 were considered for multivariable analysis. The model was reduced by backward stepwise elimination until all variables had a p-value ≤0.05. All associations were tested using likelihood-ratio tests. Site was included in the model as a fixed effect. As Kasavubu (Kinshasa) was the eldest and most experienced site, this site was considered as the reference site. This site was also used as training center for health care workers from the other sites.

**Figure 3:**
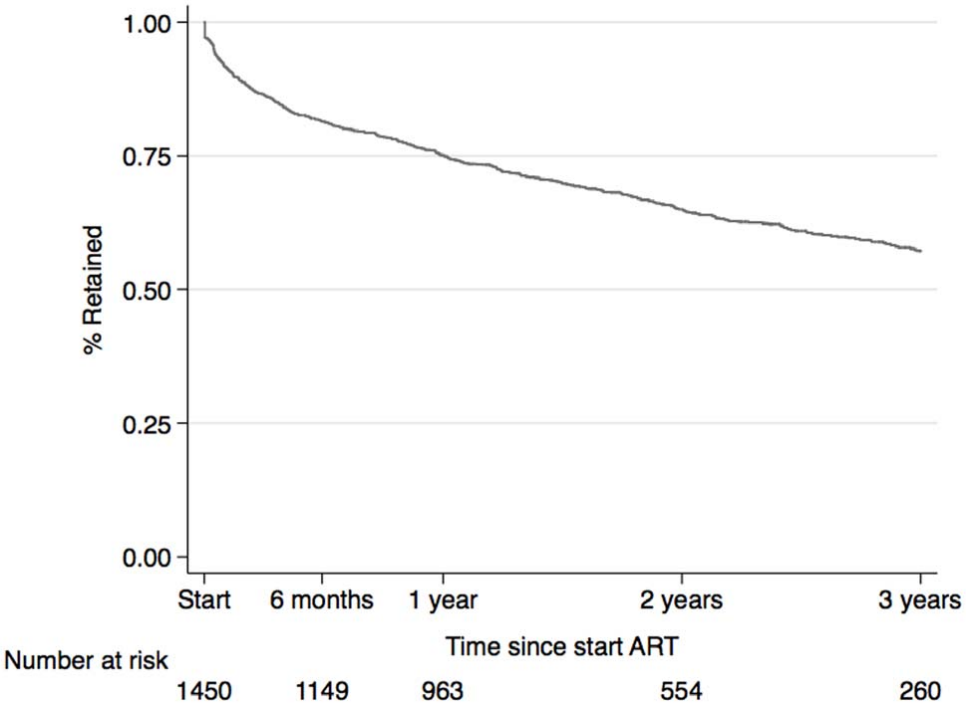
Proportion of patients discontinuing from the AmoCongo ART program over time.

**Figure 4:**
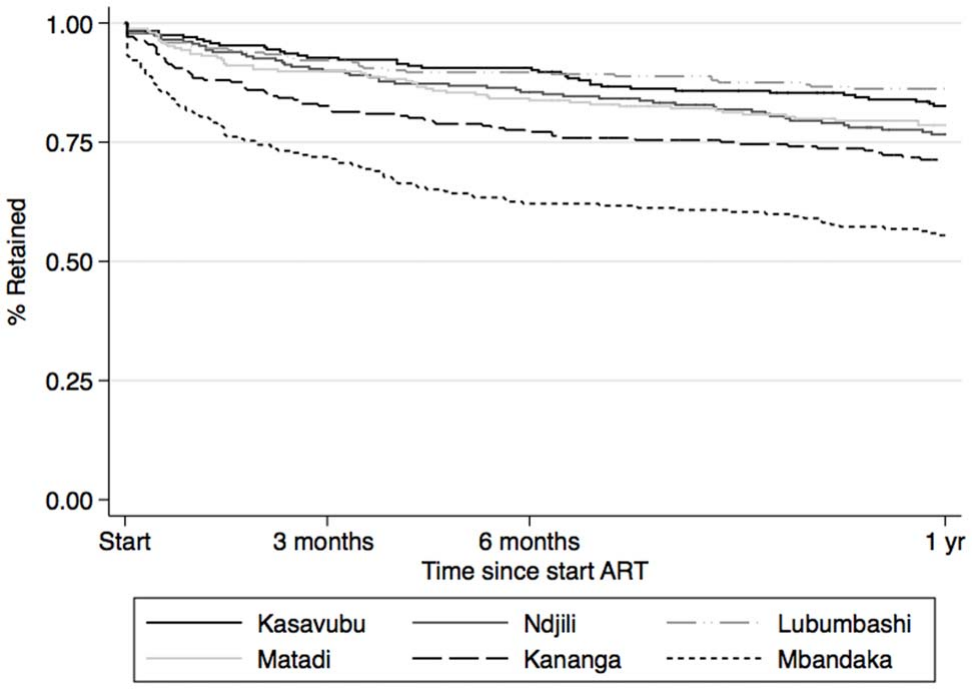
Proportion of patients discontinuing from the AmoCongo ART program at 1 year by the 6 study sites.

For variables with less than 10% missing values conditional imputation was used and for variables with at least 10% missing data the missing indicator method (in which a separate category is generated for patients with data missing for a certain predictor).

During a sensitivity analysis the association between risk factors and attrition was examined while censoring the reported deaths.

Because of the limited number of study sites, the relationship between key site characteristics and attrition was investigated as a purely explorative objective. A Cox-proportional hazards model, adjusted for clustering at the site (using a shared frailty approach) was used.

Data were entered centrally in a research database using EpiData Entry 3.1 (EpiData Association, Odense, Denmark, Europe). All statistical analyses were performed using Stata software, version 11.2. (Stata Corporation, College Station, TX, USA).

### Ethics Statement

Due to the nature of the study it was not possible and feasible to obtain informed consent from the patients whose medical charts were abstracted for the study. Data were extracted from routine records, and were analyzed anonymously.

The study protocol and data collection forms were approved by the Institutional Review Board (IRB) of the School of Public Health in Kinshasa, by the IRB of the Institute of Tropical Medicine in Antwerp and by the Ethics Committee University Hospital Antwerp.

**Table 3:** Risk factors for attrition during the first year after ART initiation.

Risk factor	Number of patients	Non-retained patients	Crude HR (95% CI)	P-value univariate analysis	HR (95% CI) Adjusted	P-value
**Total: n (%)**	1450	350 (25.5)				
**Patient baseline characteristics**						
**Year of ART initiation: n (%)**				<0.001		<0.001
2005–2006	458	62 (13.5)	1		1	
2007	461	111 (24.1)	1.80 (1.31–2.46)		1.73 (1.26,2.38)	
2008**–**2009	531	177 (33.3)	2.93 (2.19–3.92)		3.06 (2.26, 4.14)	
**Gender: n (%)**						
Male	491	130 (26.5)	1.21 (0.98–1.51)	0.085	1.32 (1.05,1.65)	0.019
Female	959	220 (22.9)	1		1	
**Age: n (%)**				0.647		
<40 years	680	169 (24.9)	1			
≥40 years	738	171 (23.2)	0.95 (0.77–1.18)			
**Civil status: n (%)**				0.116		
Single	194	59 (30.4)	1.36 (1.00–1.1.84)			
Married	675	156 (23.1)	1			
Widowed/divorced	560	131 (23.4)	1.00 (0.79–1.26)			
**Prior ART experience: n (%)**				0.868		
Yes	169	36 (21.3)	0.97 (0.68–1.38)			
No	1,257	306 (24.3)	1			
**Weight: n (%)**				0.004		0.029
Weight <50	430	135 (31.4)	1.47 (1.17–1.83)		1.33 (1.05, 1.69)	
Weight ≥50	858	183 (21.3)	1		1	
Missing	162	32 (19.8)	1.09 (0.74–1.60)		1.42 (0.95, 2.12)	
**CD4 (cells/µL) : n (%)**				0.014		NS
CD4<50	85	23 (27.1)	2.44 (1.39–4.30)			
CD4≥50<200	258	54 (20.9)	1.78 (1.11–2.84)			
CD4≥200	182	26 (14.3)	1			
Missing	925	247 (26.7)	1.52 (1.0–2.33)			
**TLC (cells/µl): n (%)**				0.195		
TLC <1200	372	104 (28.0)	1.54 (0.93–2.55)			
TLC ≥1200	74	18 (24.3)	1			
Missing	1,004	228 (22.7)	1.33 (0.81–2.19)			
**WHO stage at start: n (%)**				<0.001		<0.001
WHO stage 1**–**2	247	37 (15.0)	1		1	
WHO stage 3	847	193 (22.8)	1.33 (0.92–1.90)		1.22 (0.85, 1.76)	
WHO stage 4	129	55 (45.6)	3.12 (2.04–4.79)		2.98 (1.93, 4.59)	
Missing	227	65 (28.6)	1.33 (0.86–2.06)		1.22 (0.78, 1.91)	
**Regimen at initiation: n (%)**				0.432		
NVP based	1318	325 (24.7)	1			
EFV based	127	23 (18.1)	0.77 (0.51–1.18)			
Other	5	2 (40.0)	1.35 (0.34–5.44)			
**Distance from clinic: n (%)**				0.056		NS
<1 hour	644	126 (19.6)	1			
≥1 hour <2 hours	186	23 (12.4)	0.57 (0.36–0.89)			
≥2 hours	111	25 (22.5)	1.14 (0.72–1.82)			
Missing	509	176 (34.6)	0.86 (0.27–2.72)			
**Site: n (%)**				<0.001		<0.001
Kasavubu	236	40 (17.0)	1		1	
Ndjili	231	52 (22.5)	1.38 (0.91–2.08)		1.09 (0.72, 1.67)	
Lubumbashi	246	33 (13.4)	0.8 (0.50–1.27)		0.70 (0.44, 1.12)	
Matadi	248	52 (21.0)	1.3 (0.85–1.94)		1.03 (0.68, 1.57)	
Kananga	245	68 (27.8)	1.8 (1.23–2.70)		1.41 (0.95, 2.11)	
Mbandaka	244	105 (43.0)	3.3 (2.26–4.68)		2.85 (1.91, 4.25)	

NS: non-significant.

Data are missing when the total number of patients for a risk factor was less than 1450.

* Risk factors with p-value<0.10 during univariate analysis were considered for inclusion in the multivariable model.

**Figure 5:**
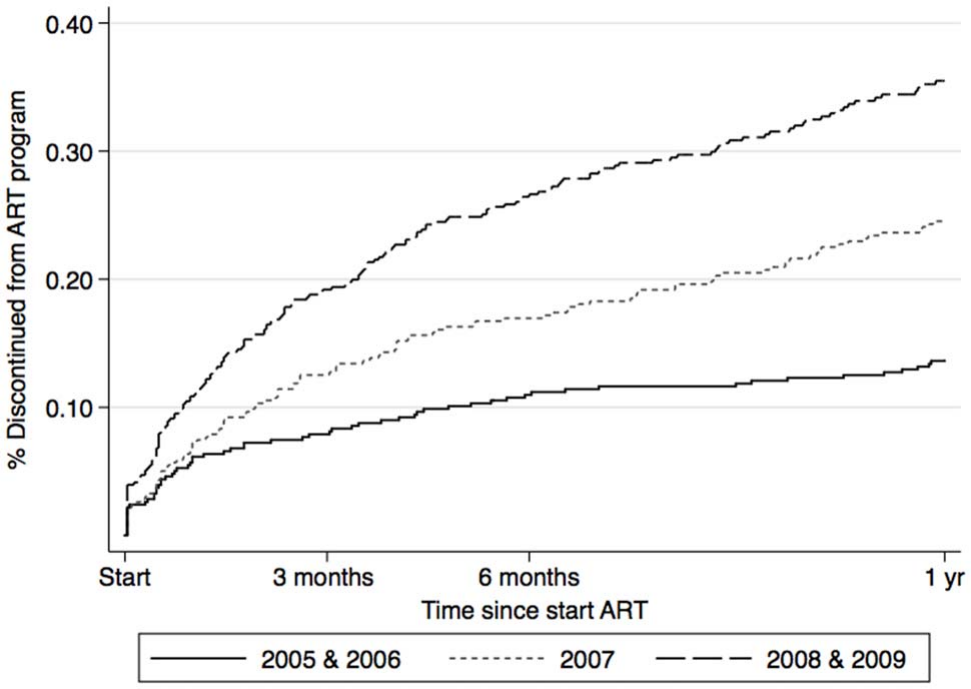
Probability of discontinuing from the AmoCongo ART program at 1 year over time by calendar year.

## Results

A total of 1,500 medical files were abstracted. Of these 50 were excluded: 2 patients were below 18 years at ART initiation, 29 had a missing date of ART initiation and 19 patients started ART before 2005. 1450 patients were included in the analysis (**Figure 2**).

### Patient Characteristics

Patients started ART in the period between January 2005 and March 2009. The number of patients initiated on ART increased over the years. Two thirds of patients were women, the mean age at start of ART was 40 years (range: 18–74), the mean weight was 56.9 kg (SD: 13.6). Only 525 patients (36.2%) had a CD4 cell count and only 446 patients (30.8%) had a total lymphocyte count before starting ART. The median CD4 cell count was 150 cells/µl (IQR: 171–238). About two thirds (67.3%) of patients were in WHO stage 3 or 4. All except 5 patients were started on a NNRTI-based regimen (**Table 1**).

### Site Characteristics

The six study sites started providing ART between 2005 and 2006. Kinshasa (with 2 sites), Lubumbashi and Matadi are urban sites, while Mbandaka and Kananga can be considered as semi-urban sites. At the time of the study, the semi-urban sites had less patients in follow-up and received fewer supervisions compared to the urban sites. Supervisions consisted of a visit of an experienced HIV physician and nurse to provide clinical support and support to the data collection process.

Four of the sites contributed data to the IeDEA Central Africa database and had access to an electronic data management system (DMS), and standardized data collection forms. However at the time of the study none of the four sites were able to use the reporting tool for patient care purposes (Table 2).

CD4 cell count was available at three of the six sites, in one site (Matadi) samples for CD4 were referred to a private lab.

Distance or time to travel to the clinic was available for 4 sites only, data on this variable was unavailable for the 2 semi-urban sites: 86% of patients were living within 2 hours traveling, 12% >2 hours and 2% had no data (data not shown).

### Retention

The overall retention rates were 81.4% (95% CI: 79.3–83.4), 75.2% (95% CI: 72.8–77.3), 65.0% (95% CI: 62.3–67.6) and 57.2% (95% CI: 54.0–60.3) at 6 months, 1 year, 2 years and 3 years respectively (**Figure 3**). The median follow-up time overall was 19 months, ranging from 15.3 months at Mbandaka to 24.2 months in Kasavubu (Kinshasa). The retention rates between sites varied between 62.1% and 90.6% at 6 months and between 55.5% and 86.2% at 1 year (**Figure 4**). During the first year 350 patients were lost from the program (**Figure 2**), 88 of them (25.1%) were reported dead and 262 (74.9%) were lost to follow-up.

### Risk Factors for Attrition

During multivariable analysis weight and WHO stage at initiation, gender, site and year of initiation remained as significant independent risk factors for attrition during the first year after ART initiation (**Table 3**). Patients who started ART in 2007 (aHR: 1.73, 95%CI: 1.26–2.38), and in 2008–2009 (aHR: 3.06, 95%CI: 2.26–4.14) were more likely to experience higher attrition (**Figure 5**) using the 2005–2006 cohort as a reference. Patients from the semi-urban sites (Kananga, aHR: 1.41, 95%CI: 0.95–2.11 and Mbandaka, aHR: 2.85, 95%CI: 1.91–4.25) experienced higher attrition compared to the cohort of Kasavubu, the center that was considered as the reference center. Male patients (aHR: 1.32, 95%CI: 1.05–1.65) were more likely to suffer from attrition. Weight below 50 kg (aHR: 1.33, 95%CI: 1.05–1.69) and a higher WHO clinical stage (aHR: 1.22, 95%CI 0.85–1.76 for stage 3 and aHR: 2.98, 95%CI: 1.93–4.59 for stage 4) were also independent risk factors.

During sensitivity analysis considering only LTFU as the outcome, the same risk factors were found as for attrition.

During the explorative analysis a high degree of collinearity between the site characteristics was found. Because of this and the limited number of study sites the model building only considered three plausible site predictors for attrition (reflecting different aspects of quality of care): number of providers, number of supervisions and CD4 availability at the health facility. The final model consisted of the same patient characteristics as the ones retained in the final fixed effect model (with similar effect sizes), and retained the number of supervisions (aHR for each additional supervision per 6 months: 0.84, 95%CI: 0.75–0.95) as site characteristic variable (data not shown).

## Discussion

As far as we know this is the first study on retention in the DRC across different sites, using a standardized data extraction tool. The overall retention rates in this local NGO supported ART program in DRC were 81.4% (95% CI: 79.3–83.4), 75.2% (95% CI: 72.8–77.3), 65.0% (95% CI: 62.3–67.6) and 57.2% (95% CI: 54.0–60.3) at 6 months, 1 year, 2 years and 3 years respectively. These low retention rates demonstrate that retention is a major challenge. Although these results seem comparable to those from other studies in sub-Saharan Africa settings [2], [8], we need to be cautious in interpreting these numbers. Whereas most of the sub-Saharan countries managed to scale up their coverage of patients on ART (with an average coverage of 36% at the end of 2009) [1], the DRC is lagging behind with a reported coverage of only 12.4% in 2010. [14] In other words, these low retention rates are even more sobering when considering the limited scaling up of ART in the DRC. Furthermore these results come from a local NGO program, which might not be representative of the situation in the public sector in the country.

There was a wide variability of retention rates between the different sites, (between 62.1% and 90.6% at 6 months and between 55.5% and 86.2% at 1 year). Retention rates were significantly lower in the 2 semi-urban sites. These 2 centers were smaller treatment centers with smaller number of health care providers and patients in HIV care. These findings contrast somehow with other studies reporting better retention in rural and decentralised sites because of greater access to services and better integration of services. [15]–[17] Probably other factors contributed to this: these centers did not have access to CD4 cell count, were not participating in the IeDEA Central Africa database (and thus did not receive the same level of support), they also received less supervisory visits. This emphasises the need for timely monitoring of key indicators (such as mortality, retention) and regular supervisory visits for training and mentoring.

Patients who started ART during the scaling up process (2007–2009) were more at risk to be lost to follow-up compared to patients starting ART during the early years (2005–2006), a finding that concurs with results from other studies. [12], [18] Rapid scaling-up may have compromised the organisation and quality of care. In the six study sites the number of patients on ART increased from 1570 in 2006 to about 7800 in 2009. This again demonstrates the need for an electronic database system with a user-friendly reporting tool enabling programs to actively and timely track patients not showing up for their scheduled appointment. An active defaulter system will also improve the reliability of mortality data of a cohort. In our study, 25% of patients not retained in the first year consisted of reported deaths, meaning that LTFU was the dominant contributor to attrition. This is consistent with findings from other ART programs in sub-Saharan Africa. [12], [19] Other studies have shown that more than 50% of patients LTFU have, in fact, died. [3], [4] Due these high mortality rates among patients LTFU after ART, and to avoid overestimation of success of ART programs, it is recommended to use attrition (death and LTFU) instead of mortality only to evaluate the success of programs. [4].

Male sex, weight below 50 kg and higher WHO stage at initiation were independent risk factors for attrition, a finding that concurs with results from other studies from sub-Saharan Africa. [9], [11], [20] Attrition was highest in the first months on ART. This can be explained by the elevated early mortality as demonstrated by others, [9], [11], [20], [21] and this in turn is due to the late presentation of patients in an advanced stage. During multivariable analysis CD4 cell count was not an independent risk factor but this could be due to the limited availability of CD4 cell count. The fact that more than half of the patients included in the study did not have a baseline CD4 cell count demonstrates the limited availability of CD4 cell count in the DRC. The limited access to ART and OI drugs for opportunistic infections (OI) in the country have resulted in persisting waiting lists for ART. WHO [22] recommends that ART should be started at a CD4 count of 350 cells/µl. However today many treatment centers in DRC are decreasing their threshold for ART even below 200 CD4 cells/µl because of insufficient access to ART. Limited CD4 cell count availability, reluctance to initiate ART without CD4 cell count (despite the WHO guidelines), the cost of laboratory tests and X-rays further hinder the scaling-up of ART in DRC.

Increasing CD4 cell count availability mainly for determining eligibility for ART, and less for clinical monitoring should be one the priorities. The DART study [23] showed that a greater public health effect would be gained from widening access to ART rather than providing routine laboratory monitoring for patients already receiving ART. In a resource-limited setting as the DRC, clinical monitoring during the first two years on treatment might be more cost-effective instead of the routine laboratory monitoring which is currently in practice.

Distance or time to travel was not an independent risk factor for attrition in our study, contrary to other studies [24] but this could be due to unavailability of this data in two of our study sites.

Limitations of the study are related to the limitations of a retrospective chart review and consist mainly of incomplete data and absence of certain variables at some of the sites. For example more than 10% of data were missing for WHO clinical stage and weight at baseline, numbers that are similar as the findings by May et al. [25] The quality of the data varied a lot amongst the sites, Forster et al found that poor quality data was associated with poor retention. [26] This was similar in this study, however the sites with lower retention did receive a lot less support in general (funding, clinical mentoring, administrative support).

This study shows that, also in DRC, retention is a major problem, while coverage of patients on ART is still very low.

With the flattening of funding for HIV care and treatment in sub-Saharan Africa, and with decreasing funding worldwide [27], [28], maximizing retention during the much needed scaling-up will even be more important.
